# PARP inhibition attenuates neuroinflammation and demyelination in a rat model of multiple sclerosis: A longitudinal [^11^C]PBR28 TSPO PET imaging study

**DOI:** 10.1016/j.neurot.2026.e01044

**Published:** 2026-08-14

**Authors:** Ge Li, Inês F. Antunes, Zihe Ming, Chunhui Wu, Bart Cornelissen, Erik FJ. de Vries

**Affiliations:** Department of Nuclear Medicine and Molecular Imaging, University Medical Center Groningen, University of Groningen, Groningen, the Netherlands

**Keywords:** Poly(adenosine diphosphate ribose) polymerase, Translocator protein, Multiple sclerosis, Neuroinflammation, Positron emission tomography

## Abstract

Poly(ADP-ribose) polymerase (PARP) plays important role in DNA repair, but is also involved in other cellular processes. PARP inhibition was suggested to suppress inflammatory signaling and has been proposed as potential treatment for neuroinflammatory disorders like multiple sclerosis. Yet, its effects on lesion-associated neuroinflammation in vivo remain insufficiently understood. We therefore evaluated whether the PARP inhibitor PJ34 can attenuate neuroinflammation and demyelination, and whether treatment response can be monitored longitudinally, using positron emission tomography (PET) with [^11^C]PBR28, a tracer for glial activation. In vitro, PJ34 was found to reduce [^11^C]PBR28 uptake and ionized calcium-binding adapter molecule 1 (IBA1) immunoreactivity in lipopolysaccharide-stimulated RAW264.7 macrophages. In vivo, focal demyelination was induced in male Sprague-Dawley rats by unilateral lysolecithin injection into the corpus callosum and striatum, followed by PJ34 or vehicle treatment and serial [^11^C]PBR28 PET imaging on days 3 and 7. PET revealed significantly lower tracer uptake in lesions of PJ34-treated animals compared to vehicle controls on day 3 (SUV_mean_ 1.21 vs. 1.75; p < 0.001) and day 7 (1.00 vs. 1.29; p < 0.001). Histological analysis confirmed reduced IBA1 immunoreactivity, smaller microglial somata, and preserved process complexity in PJ34-treated animals. Furthermore, Luxol Fast Blue staining revealed significantly greater preserved myelinated area in the PJ34-treated group at day 7 (90.9% vs. 64.8%; p < 0.001). This study demonstrates that PJ34 attenuates lesion-associated neuroinflammation and demyelination in focal white matter injury and supports longitudinal [^11^C]PBR28 PET imaging as a sensitive noninvasive biomarker of treatment response.

## Graphical abstract

**Figure:**
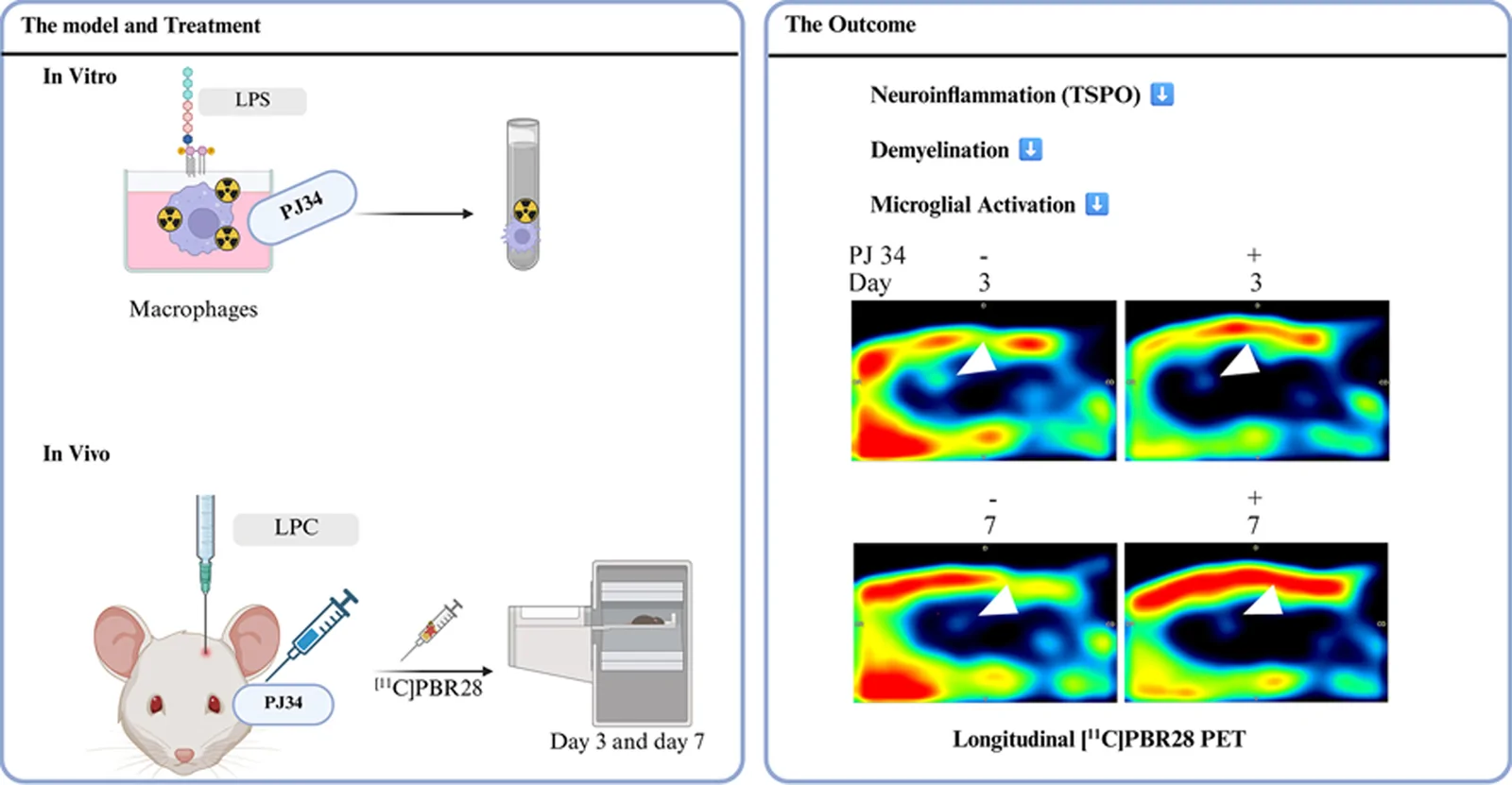

## Introduction

Neuroinflammation, inflammatory responses within the central nervous system (CNS), can be triggered by various pathological insults, including trauma, ischemia, and infection [[1], [2], [3]]. Microglia, the resident CNS immune cells, constantly survey their environment and respond rapidly to changes in inflammatory signaling. This responsiveness makes them central to both CNS homeostasis and pathological processes [[3], [4], [5], [6]]. Upon activation, microglia release cytokines, chemokines, and other mediators that drive phagocytosis and tissue repair [7,8]. In contrast to the beneficial effects of acute microglia activation, uncontrolled chronic microglial activation can lead to neuronal damage.

PARP enzymes, particularly PARP1, are essential for detecting and repairing DNA damage [[9], [10], [11]]. In conditions of chronic CNS inflammation, such as Alzheimer's disease, Parkinson's disease, and multiple sclerosis, DNA damage occurs frequently due to oxidative stress and the release of inflammatory mediators [12]. This constant damage triggers PARP1 overactivation in an attempt to repair the persistently occurring DNA breaks [13]. Overactive PARP consumes large amounts of NAD+, a crucial coenzyme for cellular energy production in mitochondria—and subsequently depletes ATP reserves. This energy drain disrupts cellular metabolism, leading to “PARP-induced cell death” or parthanatos, a form of programmed cell death distinct from apoptosis [14].

In the context of neuroinflammation, excessive PARP activation can amplify inflammatory responses by inducing the expression of pro-inflammatory genes, which activate glial cells. Activated microglia release cytokines and reactive oxygen species (ROS) that sustain a vicious cycle of DNA damage and PARP overactivation, worsening neuronal injury [15]. Moreover, PARP overactivation affects blood-brain barrier integrity, allowing peripheral immune cells to infiltrate the CNS, thereby intensifying inflammation and neurodegeneration [16,17]. Recent research suggests that targeting PARP overactivation could potentially protect neurons by conserving cellular energy and reducing inflammatory signaling [18,19]. PARP inhibitors, currently used in cancer therapy, could be explored as potential neuroprotective agents in experimental models of neurodegenerative diseases. This may offer new therapeutic avenues for mitigating neuroinflammation-driven damage and progression in CNS disorders like multiple sclerosis.

The aim of this study was to investigate the effect of PARP inhibition on neuroinflammation in the demyelinated lesion of the lysolecithin rat model for multiple sclerosis. For this study, PJ34 was selected as the PARP inhibitor, as it is able to pass the intact blood–brain barrier. We hypothesized that PJ34 would attenuate microglial activation and demyelination. The effect of treatment was evaluated using PET and postmortem histology. PET imaging enables in vivo quantification of glial activation using TSPO ligands. [^11^C]2-[6-(4-methoxyphenoxy)pyridin-3-yl]-N-(pyridin-3-ylmethyl)acetamide ([^11^C]PBR28) is a PET tracer that binds with high-affinity to TSPO. Activation of microglia leads to overexpression of TSPO, which can be detected with [^11^C]PBR28 PET. Thus, [^11^C]PBR28 PET was used for detecting small neuroinflammatory changes in response to treatment.

## Materials and Methods

### Radiochemistry

[^11^C]PBR28 was synthesized from cyclotron-produced [^11^C]CH_4_ via [^11^C]CH_3_I methylation of the desmethyl precursor. A detailed description of the radiosynthesis, purification and quality control of [^11^C]PBR28 is provided in the supplemental material under “Radiochemistry”.

### Cell cultures

The murine RAW264.7 macrophage cell line was obtained from the American Type Culture Collection (TIB-71, ATCC). The cell line was tested for the absence of mycoplasma according to the manufacturer's instructions (MycoAlert ® Mycoplasma Detection Kit, LT07-318, Lonza Bioscience). Cells beyond passage 20 were excluded from the experiments to ensure phenotypic stability and experimental reproducibility. The RAW264.7 cell line was cultured in Dulbecco's Modified Eagle's Medium (Gibco™ DMEM, 11584486, Fisher Scientific) supplemented with 10% fetal bovine serum (Gibco™ Fetal Bovine Serum, 17479633, Fisher Scientific) at 37 °C with 5% CO_2_.

### In vitro studies

Lipopolysaccharide (LPS, L2630, Sigma) was used to activate macrophage cells. To determine the required concentration of LPS, RAW 264.7 macrophage cells were seeded at 10^5^ cells per well in a 24-well plate. The next day, cells were exposed to LPS (100 or 1000 ng/mL in PBS), or vehicle, for 24 h [20]. Following stimulation, the medium was removed and cells were washed with PBS. Subsequently, fresh medium containing 50–100 kBq of [^11^C]PBR28 was added to the medium and incubated at 37 °C for 30 min. After incubation, the cells were washed three times with cold phosphate-buffered saline (PBS, 10010023, Gibco) and lysed with 250 μL of 1 M NaOH (901915, Sigma) at room temperature for 10 min. The amount of radioactivity associated with the cells was counted in a γ-counter. In parallel experiments, cell numbers were quantified in identically treated groups. Tracer uptake was normalized for cell number and tracer dose, and to the average uptake in control cells in the same experimental replicate.

To examine the effect of the PARP inhibitor PJ34 (AA00C6EK, AA BLOCKS INC.) on macrophage activation, 10^5^ RAW 264.7 cells were exposed to different concentrations of PJ34 (1, 10, or 20 μM, or vehicle control) for 1 h. LPS (100 ng/mL) or vehicle was added, and the cells were incubated at 37 °C for 24 h. On the following day, cells were washed and fresh medium was added. [^11^C]PBR28 (50–100 kBq) was added and incubated at 37 °C for 30 min. Subsequently, cells were washed and processed as described above.

### Immunocytochemistry

RAW 264.7 cells were seeded at 1 × 10^4^ cells per well on an 8-well chamber slide. The following day, RAW 264.7 cells were exposed to PJ34 (5 μM, 10 μM or 20 μM) [20] or PBS (vehicle control), 1 h prior to the incubation with 100 ng/mL of LPS. After incubation for 24 h, the cells were fixed with 4% paraformaldehyde for 15 min, permeabilized with 0.1% Triton X-100 for 10 min, and blocked with 5% bovine serum albumin for 30 min. Primary antibody IBA1 (019–19741, Wako, 1:500) was applied overnight at 4 °C, followed by a Goat Anti-Mouse IgG H&L Alexa Fluor® 488 fluorescent secondary antibody (ab150113, Abcam, 1:1000) for 1 h at room temperature. Nuclei were counterstained with DAPI and images were captured using a confocal microscope (Leica SP8). Micrographs were analyzed using ImageJ software (NIH, USA), and the relative fluorescence intensity was calculated as the ratio of IBA1 to DAPI signal.

### Western blot analysis

Whole-cell lysates were prepared from RAW264.7 macrophages using RIPA Lysis and Extraction Buffer (89900, Thermo Fisher Scientific). Protein concentrations were determined using a BCA protein assay. Equal amounts of protein (20 μg per lane) were denatured and separated on 4–12% gradient SDS–PAGE gels using an Invitrogen Mini Gel Tank (15320424, Thermo Fisher Scientific), followed by transfer onto PVDF membranes.

The membranes were blocked with 5% bovine serum albumin in Tris-buffered saline containing 0.1% Tween-20 (TBST) for 1 h at room temperature and subsequently incubated overnight at 4 °C with a rabbit monoclonal antibody against PARP1 (9532S, Cell Signaling Technology, 1:1000) or a mouse monoclonal antibody against β-actin (ab8224, Abcam, 1:1000). After washing with TBST 3 times for 5 min, membranes were incubated for 1 h at room temperature with goat anti-rabbit IgG H&L (HRP) (ab205718, Abcam, 1:5000) for PARP1 or goat anti-mouse IgG H&L (HRP) (ab205719, Abcam, 1:5000) for β-actin. After washing, protein bands were visualized using an enhanced chemiluminescence substrate and imaged. Three independent RAW264.7 cell preparations (n = 3) were analyzed, with two replicate wells included for each preparation. Lysates from all six wells were analyzed on the same membrane.

### Animal model

All animal experiments were approved by the National Committee on Animal Experiments of the Netherlands (CCD license no. AVD1050020198648) and the local animal welfare committee of the University Medical Center Groningen (protocol no. 198648-02-006). All animals (Sprague-Dawley rat, Envigo) were housed in conventional cages in groups of up to five until surgery and subsequently in individual IVC cages until the end of the experiment. Animals were housed in an artificial day-night cycle facility with *ad libitum* access to food and water.

Demyelinating lesions were induced in male Sprague–Dawley rats (8–10 weeks of age) by stereotactic injection of 1% lysolecithin (l-α-lysophosphatidylcholine; Sigma-Aldrich) dissolved in sterile saline. For analgesia, buprenorphine (0.01 mg/kg, s.c.) and marcaine (0.25%, 100 μL, local) were administered prior to surgery. Animals were anaesthetized with isoflurane (5% for induction, 2% for maintenance) and placed in a stereotactic frame. A volume of 3 μL 1% lysolecithin in saline was injected into the corpus callosum and 2 + 2 μL into the striatum at a rate of 0.1 μL/min using a Hamilton syringe. These injections were performed −0.3 mm anterior and −3.0 mm lateral relative to Bregma at a ventral depth of −3.0, −4.2, and −5.0 mm, respectively. Following each injection, the needle was left in place for 5 min to prevent backflow and then slowly withdrawn.

The needle was cleaned with 70% ethanol and saline between injections to ensure smooth and unobstructed delivery. Animals were monitored postoperatively until full recovery. The bodyweight of the rats was monitored every day after surgery.

### PARP inhibitor treatment

**Fig. 1:**
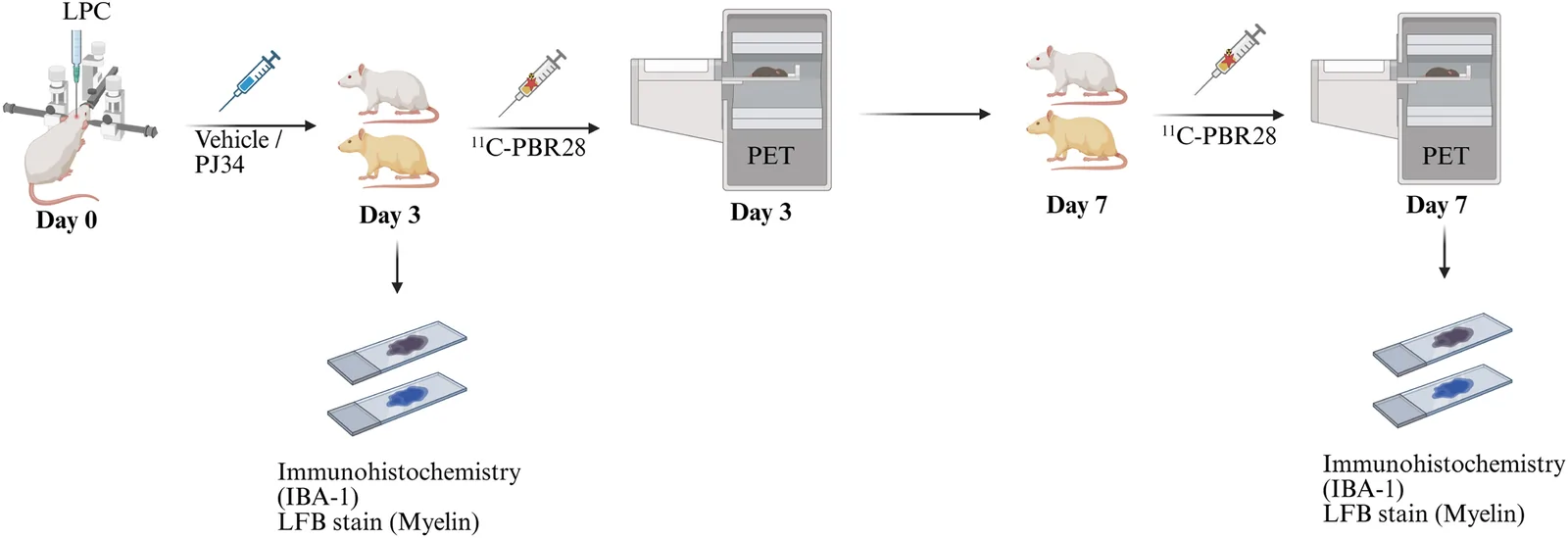
Animal experimental workflow.

Animals were randomly divided into 2 groups of 13 animals (1) vehicle treated: 100 μL of saline administered every 12 h by intraperitoneal injection and (2) PJ34 treated: 20 mg/kg bodyweight PJ34 dissolved in 100 μL of saline solution administered every 12 h by intraperitoneal injection. Treatment was started around 12 h after the surgery and was continued for 3 days. In each group, 11 animals were subjected to PET imaging on day 3. Three of these animals were terminated on day 3 for postmortem analysis, whereas the remaining 8 animals were scheduled for PET imaging on day 7. In each group, remaining 2 animals were terminated for postmortem analysis on day 3, without having a PET scan, resulting in a total sample size of 5 animals per group for postmortem analysis on day 3. The schematic in Fig. 1 summarizes the study design.

### PET imaging

[^11^C]PBR28 PET imaging was performed 3 and 7 days after lysolecithin administration. Animals were anesthetized with 2% isoflurane in oxygen. [^11^C]PBR28 (33.2 ± 2.7 MBq; molar activity 129 ± 37 GBq/μmol; ≤1 mL) was administered intravenously and animals were allowed to wake up. After 40 min, the animals were anaesthetized again and positioned in a dedicated small animal PET/CT scanner (Inveon, Siemens). A transmission scan was acquired using a ^57^Co source to correct for attenuation and scatter. Acquisition of a 15-min static emission scan was started 45 min after tracer injection. Each scan, two animals (one from each group) were scanned simultaneously.

Eleven animals per treatment group were scheduled for PET imaging on day 3. Due to tracer production, cannulation, or scanner-related technical failures, three acquisitions per group did not yield evaluable data, resulting in eight evaluable scans per group on day 3. Eight animals per group were scheduled for a second PET scan on day 7. One acquisition per group did not yield evaluable data on day 7 because of a technical failure, resulting in seven evaluable scans per group at this time point.

Because the technical failures did not necessarily affect the same animals at both time points, the availability of PET data was unbalanced. In the vehicle group, five animals had evaluable scans at both time points, three had evaluable data only on day 3, two had evaluable data only on day 7, and one animal had no evaluable PET data at either time point. In the PJ34 group, four animals had evaluable scans at both time points, four had evaluable data only on day 3, and three had evaluable data only on day 7. All available data were included in the analysis. SUV values and data availability of individual animals at each time point are provided in Supplementary Table S1.

### PET image reconstruction and analysis

Emission sinograms were iteratively reconstructed after being normalized, corrected for scatter, attenuation and decay of radioactivity. The 15-min static scan was reconstructed (OSEM2D with 4 iterations) as a single frame.

Images were analyzed using PMOD (version 4.1) software. First, a tracer-specific rat brain template was created by averaging the manually co-registered individual PET images. Each individual PET image was then registered to this template for group analysis. Volume of interest (VOI) (a 3 mm diameter sphere centered at the injection site) was defined in the template space based on the stereotactic injection coordinates and verified using an anatomical reference template (MRI). A reference VOI was generated by flipping the ipsilateral VOI to the opposite hemisphere. For each VOI, the radioactivity concentration was determined and corrected for the injected dose and body weight to give the standardized uptake value (SUV). A Gaussian post-filter (2 mm FWHM) was applied to the PET images for representation purposes only.

### Immunohistochemistry and myelin staining

Animals were assigned to either the vehicle or PJ34 treatment group (n = 13 per group). In each treatment group, 11 animals constituted the PET cohort. All animals in the PET cohort were scheduled for [^11^C]PBR28 PET imaging on day 3 after LPC administration. Three animals per group were assigned to the day 3 endpoint and were euthanized after the day 3 imaging time point, whereas the remaining eight animals per group were scheduled for longitudinal PET imaging on days 3 and 7. In addition, two animals per treatment group constituted a day 3 histology-only cohort. These animals underwent the same LPC lesion induction and treatment regimen as the PET cohort but did not undergo PET imaging. They were euthanized on day 3 for postmortem analyses. Consequently, the day 3 histological analyses included five animals per treatment group: three animals from the PET cohort and two animals from the histology-only cohort. The histology-only animals were not included in any PET or SUV analyses.

Animals remained anesthetized with 2% isoflurane in oxygen. A midline thoracotomy was performed to expose the heart. A perfusion needle was inserted into the left ventricle, and the right atrium was incised to allow drainage. Transcardial perfusion was conducted using approximately 100 mL of cold 0.9% saline, followed by 100 mL of freshly prepared 4% paraformaldehyde in phosphate-buffered saline (PBS). Following perfusion, brains were harvested, post-fixed in 4% paraformaldehyde for 24 h at 4 °C and then processed for paraffin embedding using standard dehydration and infiltration procedures. Coronal brain sections of 4 μm thick were cut and mounted on slides.

For immunohistochemistry, sections were deparaffinized, rehydrated (Xylene 10 min; Xylene 5 min; 100% ethanol 5 min; 96% ethanol 5 min; 70% ethanol 5 min; demineralized water 5 min; then washed two times in PBS for 5 min), and subjected to antigen retrieval with sodium citrate and citric acid. After blocking with blocking buffer (PBS supplemented with 5% BSA and 0.1% Triton X-100) for 30 min at room temperature, sections were incubated with rabbit anti-IBA1 for one night at 4 °C (019–19741, Wako, 1:500 diluted in blocking buffer), followed by Alexa Fluor 488 conjugated goat anti-rabbit IgG (ab150077, Abcam, 1:1000). Nuclei were counterstained with DAPI. Images were acquired using Leica Thunder or Leica SP8 confocal microscope. Three sections per animal spanning the injection site were analyzed. ROIs were drawn based on the injection site or the contralateral site as a control region, and the relative IBA1 signal was quantified as the ratio of IBA1 to DAPI fluorescence using Fiji, an open-source ImageJ-based image analysis software package. Microglial morphology in the lesion area was analyzed in Fiji. For soma size analysis, individual IBA1-positive cells (3 sections per animal, 5 animals) with clearly identifiable cell bodies were selected, and the soma area was manually outlined and measured using the freehand selection tool. For Sholl analysis, isolated IBA1-positive cells were selected and concentric circles centered around the soma were applied using the Sholl Analysis plugin to quantify process complexity [21]. The number of intersections of the concentric circles with the cell membrane was recorded for increasing radial distances from the soma. More intersections represent a more complex microglial structure. Analyses were performed on cells from the lesion area, and mean values were calculated per animal (n = 5) for statistical analysis.

For Luxol Fast Blue (LFB) staining, paraffin-embedded brain sections (10 μm) were deparaffinized in xylene (3 × 10 min), rehydrated with ethanol (100% and 95%), and stained overnight in 0.1% LFB solution (0.2 g LFB in 200 mL 95% ethanol with 1 mL 10% acetic acid, filtered) at 50 °C. After rinsing in 95% ethanol and tap water, differentiation was performed using 0.05% lithium carbonate (20 s) followed by 70% ethanol (3 changes; 1–10 min) until gray matter appeared colorless. Sections were then washed, dehydrated in ethanol, cleared in xylene, and cover slipped. LFB-stained sections were imaged using an EVOS XL Core microscope (Invitrogen). Myelin content was quantified using Fiji/ImageJ by measuring the LFB-positive area within manually defined lesion ROIs. The percentage of myelinated area was calculated as the LFB-positive area divided by the total ROI area. Three sections per animal were analyzed, and the mean value was used for statistical analysis.

### Statistical analysis

All data were obtained at least in triplicate. All statistical analyses were performed using GraphPad Prism version 9.5.0 or 10.1.0, SPSS version 30.0.0.0 (171) or Fiji. Differences between groups were analyzed for statistical significance using a 1-way ANOVA followed by Tukey's post hoc test (multiple groups). The PET data constituted an unbalanced repeated-measures dataset because some animals had an evaluable scan at only one time point. The generalized estimating equation (GEE) model was fitted using all available observations, with animal identity specified as the repeated-measures subject. Animals with an evaluable scan at only one time point contributed data to that time point, whereas animals without any evaluable PET acquisition did not contribute to the PET analysis. Missing PET values were not imputed. Data are presented as the mean ± sample standard deviation across animals at each time point. Results are reported as mean ± standard deviation. Differences were considered significant when p < 0.05.

## Results

### PJ34 attenuates LPS induced activation of RAW 264.7 cells

**Fig. 2:**
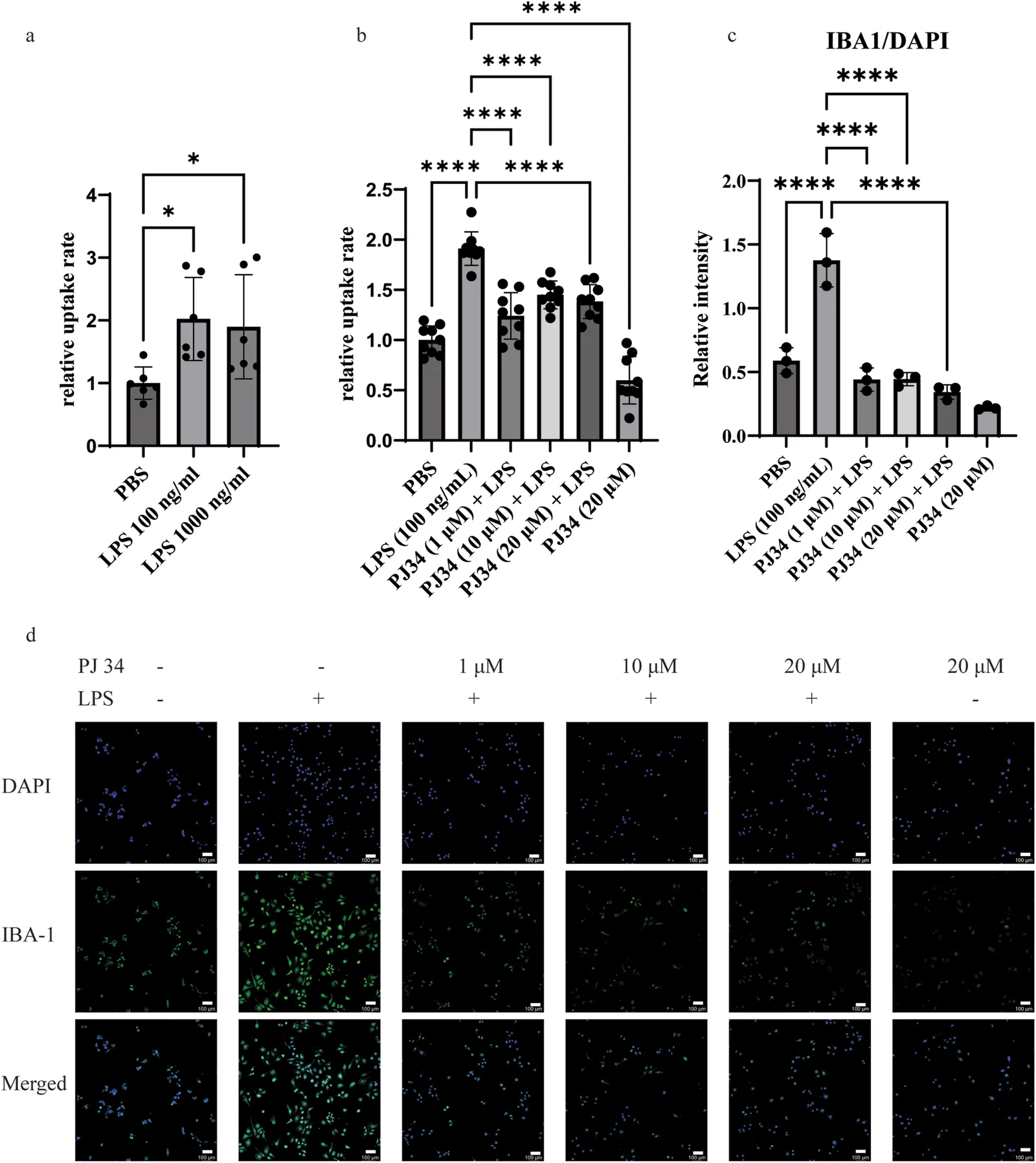
PJ34 attenuates LPS-induced activation of RAW 264.7 cells. (a) Quantification of relative uptake of [^11^C]PBR28 in RAW 264.7 cells treated with PBS or LPS at 100 or 1000 ng/mL for 24 h. (b) Quantification of the effect of PJ34 treatment (1, 10, or 20 μM) on [^11^C]PBR28 uptake in LPS (100 ng/mL)-activated RAW 264.7 cells. (c) Quantification of IBA-1 fluorescence intensity, relative to DAPI signal. (d) Representative immunofluorescence images of RAW 264.7 cells stained for DAPI (blue) and IBA-1 (green) after treatment with LPS and/or PJ34. Scale bar: 100 μm ∗p < 0.05, ∗∗p < 0.01, ∗∗∗p < 0.001, ∗∗∗∗p < 0.0001.

Western blot analysis confirmed full-length PARP1 protein expression in three independent RAW264.7 cell cultures (n = 3), with β-actin serving as the loading control (Fig. S3). Having confirmed the presence of PARP1 in this cellular model, we assessed whether PARP inhibition by PJ34 could suppress LPS-induced macrophage activation. RAW264.7 cells were first stimulated with LPS, and [^11^C]PBR28 uptake was measured as a surrogate marker of macrophage activation. Treatment with 100 ng/mL LPS significantly increased tracer uptake relative to vehicle (PBS) treated cells (p = 0.02; Fig. 2a). Increasing the LPS dose to 1000 ng/mL did not significantly increase [^11^C]PBR28 uptake any further. Having confirmed that LPS robustly increases [^11^C]PBR28 uptake, we next examined whether PJ34 could counteract this activation. Pre-treatment with PJ34 at concentrations of 1, 10, or 20 μM significantly reduced LPS-induced uptake of [^11^C]PBR28 (p < 0.0001 for all comparisons with cells treated with LPS alone; Fig. 2b). Nevertheless, uptake in the PJ34 + LPS groups remained above PBS control levels, suggesting partial rather than complete normalization of LPS-induced tracer uptake. PJ34 (20 μM) also decreased [^11^C]PBR28 uptake in unstimulated control cells (p = 0.005). No significant differences were observed between the three PJ34 concentrations. To confirm that the suppression of activation observed by [^11^C]PBR28 uptake corresponded to changes at the protein level, immunocytochemistry was performed to assess the expression of IBA-1, a macrophage activation marker. LPS significantly increased IBA1 fluorescence intensity (p < 0.0001), consistent with activation, while co-treatment with PJ34 reduced IBA1 fluorescence intensity to the level of control cells (p < 0.0001, Fig. 2c and d). PJ34 treatment (20 μM) also decreased IBA1 expression in untreated control cells, as determined by immunocytochemistry quantification.

Together, these results demonstrate that PJ34 effectively attenuates LPS-induced functional and phenotypic activation of RAW 264.7 macrophages.

### [^11^C]PBR28 PET imaging to monitor modulation of microglial activation by PARP inhibition in the lysolecithin model of MS

To assess the effects of PARP inhibition on neuroinflammation, we employed [^11^C]PBR28 PET imaging to monitor microglial activation in a LPC-induced demyelination model in rats. Individual ipsilateral lesion and contralateral SUV_mean_ values for every evaluable scan, together with group mean and SD, are provided in Table S1.

**Fig. 3:**
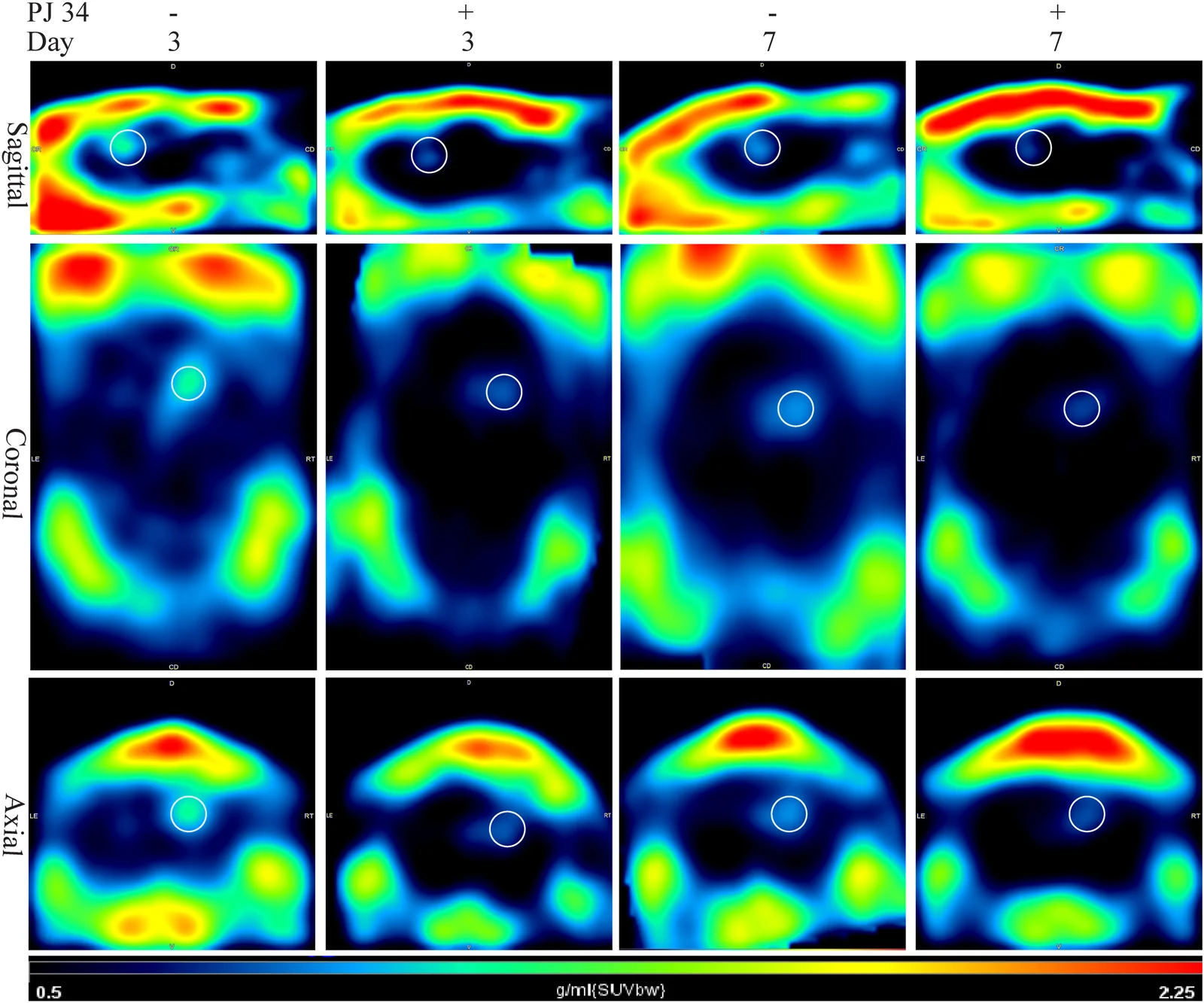
Static [^11^C]PBR28 PET scans were acquired on Day 3 and Day 7 after LPC injection to assess TSPO expression as a marker of microglial activation. Each scan was acquired over 15 min, starting 45 min after tracer injection. The white circle indicate the approximate locations of the lesion VOIs.

**Fig. 4:**
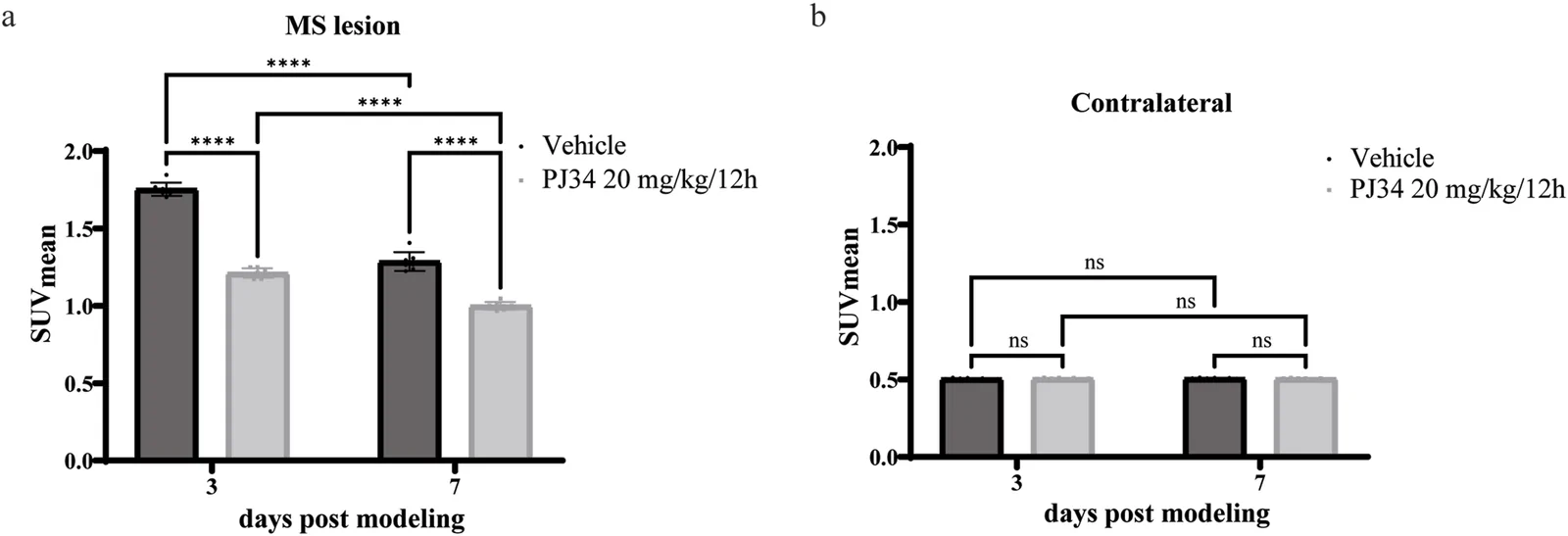
Quantification of [^11^C]PBR28 uptake in the lysolecithin rat model for MS. (a) In the ipsilateral hemisphere, PJ34 significantly reduced uptake at both time points. (b) Contralateral hemisphere. ∗∗∗∗p < 0.0001; ns: not significant.

In vehicle-treated animals, [^11^C]PBR28 uptake was significantly higher in the ipsilateral hemisphere compared with the contralateral side at both day 3 (1.75 ± 0.04 vs. 0.505 ± 0.003, p < 0.001; n = 8) and day 7 (1.29 ± 0.06 vs. 0.507 ± 0.005, p < 0.001; n = 7; Fig. 3, Fig. 4). These results are consistent with a peak in LPC-induced glial activation at the injection site – but not in the contralateral hemisphere. The results indicated that glial activation at the injection site had partially resolved on day 7. PJ34 treatment significantly reduced [^11^C]PBR28 uptake at the injection site both on day 3 (1.21 ± 0.03 vs. 1.75 ± 0.04, p < 0.001; n = 8) and on day 7 (1.00 ± 0.03 vs. 1.29 ± 0.06, p < 0.001; n = 7), demonstrating that PARP inhibition attenuates glial activation, as detected by [^11^C]PBR28 PET. Treatment with PJ34 did not induce any significant differences in tracer uptake in the contralateral hemisphere at either time point (p > 0.5).

### Postmortem analysis confirms that PJ34 reduces microglial activation and shows that PJ34 preserves myelin integrity in the LPC-induced lesion

**Fig. 5:**
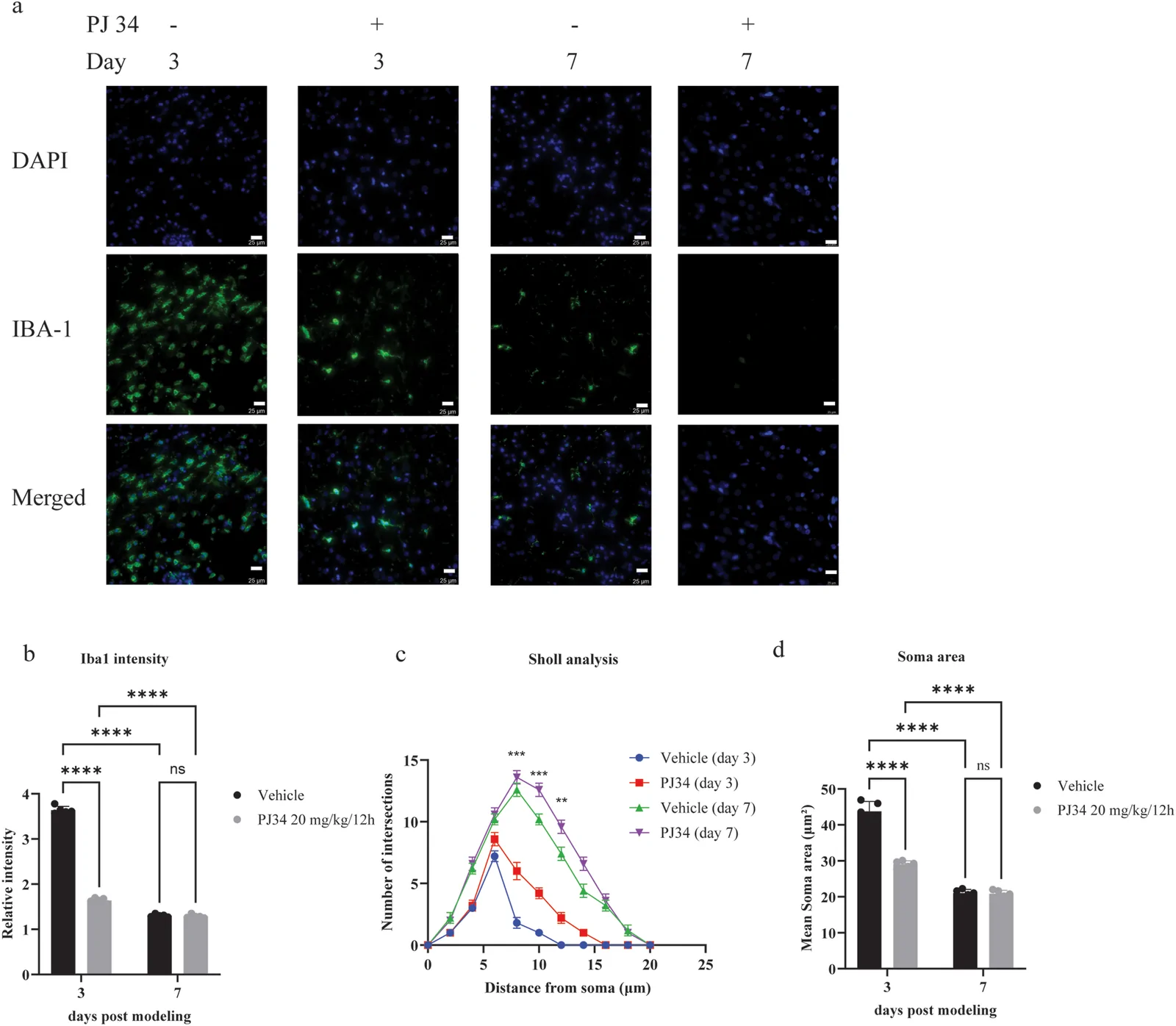
PJ34 reduces IBA1 expression and preserves resting microglial morphology following LPC induced demyelination. (a) Representative immunofluorescence images of rat brain sections stained for IBA1 (green) and counterstained with DAPI (blue, nuclei) at day 3 and day 7 after LPC injection, with or without PJ34 treatment. Scale bars: 25 μm. (b) Quantification of IBA1 fluorescence intensity (n = 5) expressed as relative intensity to DAPI. (c) Sholl analysis (n = 5) of microglial morphology assessing the number of intersections at increasing radial distances from the soma. (d) Quantification of mean microglial soma area (μm^2^) (n = 5). ∗∗p < 0.01, ∗∗∗∗p < 0.0001; ns: not significant.

To confirm microglial activation in response to LPC-induced demyelination and the modulatory effect of PJ34, IBA1 immunohistochemical staining was performed on brain sections (Fig. 5). At day 3 after LPC administration, vehicle-treated animals displayed robust IBA1 immunoreactivity at the lesion area, with microglia exhibiting amoeboid morphology characterized by enlarged somata and thickened processes (Fig. 5a). Quantification confirmed significantly higher relative IBA1 fluorescence intensity in vehicle-treated rats (3.66 ± 0.11) compared with PJ34-treated animals (1.48 ± 0.08; p < 0.0001, Fig. 5b). By day 7, relative IBA1 fluorescence intensity in the vehicle group was significantly reduced (1.29 ± 0.02; p < 0.0001) and was comparable with the IBA1 intensity in the PJ34-treated group (1.26 ± 0.02; p = 0.96). These data indicate that PJ34 significantly reduced microglial activation at day 3. To further evaluate the morphological changes associated with microglial activation, we performed Sholl analysis to quantify process complexity and measured the soma area of IBA1-positive cells. At day 3 post-injection, microglia in the vehicle group displayed a severe loss of process complexity, typical of the amoeboid phagocytic state, with the number of intersections rapidly dropping to zero beyond 10 μm from the soma (Fig. 5c). Conversely, treatment with PJ34 significantly preserved microglial ramification, exhibiting a broader intersection profile and maintaining processes that extended up to 14 μm from the soma. By day 7, microglia in both the vehicle and PJ34-treated groups demonstrated highly ramified morphologies with intersections occurring up to 18 μm (peak intersection at 8 μm), indicating a natural resolution of the acute inflammatory state and confirming that early PJ34 intervention did not impair long-term morphological recovery. Consistent with these observations, quantification of microglial soma area (Fig. 5d) revealed pronounced cellular hypertrophy in vehicle-treated animals at day 3 (43.8 ± 1.2 μm^2^). In contrast, PJ34-treated animals exhibited significantly smaller soma area at the same time point (29.0 ± 0.4 μm^2^; p < 0.0001), indicating an attenuation of the amoeboid transformation. At day 7, the soma areas in both groups markedly decreased to sizes characteristic of resting microglia (vehicle: 21.1 ± 0.5 μm^2^; PJ34: 20.9 ± 0.4 μm^2^), with no significant difference observed between the two groups (p > 0.05). Taken together, these morphological parameters robustly demonstrate that PJ34 effectively suppresses the acute activation of microglia.

**Fig. 6:**
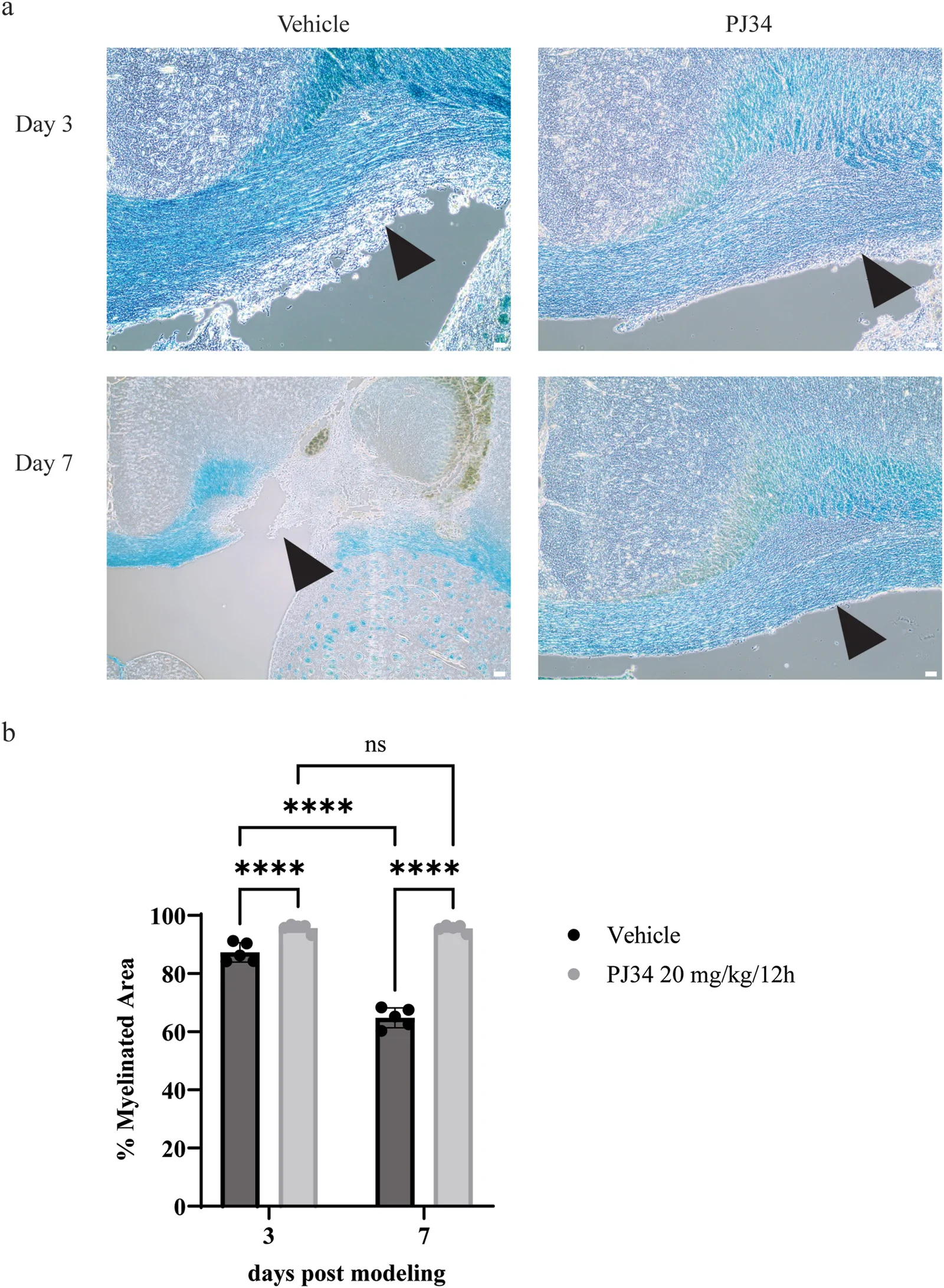
PJ34 preserves myelin integrity in an LPC-induced demyelination model. (A) Representative Luxol Fast Blue (LFB)–stained coronal brain sections from rats collected at 3 and 7 days post-LPC injection (corpus callosum is shown in this figure). Scale bar: 250 μm. Images are representative of n = 5 per group. (B) Quantification of the percentage of myelinated area (% Myelinated Area) in corpus callosum. ∗∗∗∗p < 0.0001; ns: not significant.

To assess the extent of demyelination and the effect of PJ34 on white matter preservation, we performed Luxol Fast Blue (LFB) staining on coronal brain sections (Fig. 6, suppl. Fig. S2). At day 3 after LPC administration, vehicle-treated animals displayed partial loss of LFB staining in the lesion area, indicative of early-stage demyelination (87.3 ± 4.4% myelinated area). By day 7, myelin loss was more extensive, with a clearly demarcated pale region in the corpus callosum and adjacent white matter, reflecting substantial structural damage (64.8 ± 4.1% myelinated area). In contrast, PJ34-treated rats showed improved myelin preservation. At day 3, PJ34-treated animals showed a higher percentage of myelinated area than vehicle-treated animals (93.3 ± 2.0%; p = 0.025 vs. vehicle day 3). By day 7, PJ34-treated brains retained substantially more LFB-positive myelin staining than vehicle controls (90.9 ± 3.4%; p < 0.001 vs. vehicle day 7). Furthermore, no significant difference in myelin preservation was observed between day 3 and day 7 in the PJ34-treated group (p = 0.221), suggesting sustained preservation of myelin staining over time. These findings indicate that PJ34 attenuates LPC-induced demyelination and supports structural preservation in the lesion area.

## Discussion

Neuroinflammation, prominently mediated by activated microglia, plays a pivotal role in the pathogenesis and progression of multiple sclerosis (MS). In the current study, we evaluated the effects of the PARP inhibitor PJ34 in an LPC-induced rat model of focal white matter injury using longitudinal [^11^C]PBR28 TSPO PET imaging combined with histopathology. Our findings show that PJ34 reduced lesion-associated TSPO PET signal, decreased IBA1 immunoreactivity, preserved a more ramified microglial morphology, and attenuated demyelination. Together, these data support a protective effect of PARP inhibition on lesion-associated neuroinflammation and tissue injury during both the acute and subacute phases of lesion development.

Our in-vitro studies, showed that RAW264.7 macrophages express PARP1, that LPS can activate these cells, as shown by increased [^11^C]PBR28 binding and IBA-1 expression, and that PARP1 inhibitor PJ34 can moderate the LPS-induced activation. These results are in line with several other in-vitro studies, which have shown that LPS activates PARP1 and protein PARylation in RAW264.7 macrophages and that pharmacological inhibition with PJ34 or PARP1 silencing attenuates PARP1-dependent inflammatory signaling [[22], [23], [24]].

In our in-vivo study, LPC produced robust ipsilateral glial activation and demyelination, confirming the expected inflammatory and structural response of this model. In vehicle-treated animals, [^11^C]PBR28 PET demonstrated sustained ipsilateral TSPO upregulation, which corresponded histologically to dense microglial infiltration and amoeboid morphology in LPC-lesioned areas. Amoeboid microglia, defined by retracted processes and hypertrophic soma, are hallmarks of a reactive, phagocytic phenotype associated with cytokine release and tissue damage [25]. These results suggest that [^11^C]PBR28 PET might be used as a highly reliable, non-invasive biomarker for tracking microglial activation and neuroinflammation dynamics in this focal demyelination model. Our findings are consistent with the established pathophysiology of the LPC model, in which microglial activation peaks during the first few days after lesion induction, while demyelination progresses over the first week [26]. Similar time courses of glial activation have been reported in previous LPC studies, confirming that the model in our hands reproduces the expected dynamics of inflammation and myelin loss. Thus, our control data validate the utility of this model for studying the interplay between microglial activation and demyelination.

The anti-inflammatory effects of PJ34 observed in our study via PET were supported by IBA1 immunostaining. PJ34 treatment reduced [^11^C]PBR28 uptake as measured with PET and significantly decreased IBA1 immunoreactivity, with microglia displaying a more ramified morphology indicative of homeostatic surveillance rather than pro-inflammatory activation. Thus, both PET and immunohistochemistry provided concordant evidence for PJ34-mediated suppression of microglial activation, both quantitatively in terms of reduced TSPO expression (PET) and IBA1 intensity and qualitatively in terms of phenotype. These findings align with previous reports showing that PARP inhibition exerts anti-inflammatory effects in models of CNS injury and neurodegeneration. PJ34 is known to cross the BBB effectively, which may explain its ability to moderate neuroinflammation, in contrast to PARP inhibitors with limited CNS availability [[27], [28], [29]]. Mechanistically, PARP-1 promotes inflammatory gene expression by co-regulating NF-κB- and AP-1-dependent transcriptional programs, thereby enhancing the production of pro-inflammatory mediators [[30], [31], [32]]. By inhibiting PARP-1, PJ34 may dampen this pathway and promote a more balanced or anti-inflammatory microglial state, aligning with the reduction in TSPO expression and glia activation observed in this study.

In addition to its effect on inflammatory activity, PJ34 reduced demyelination at day 7, indicating protection against lesion-associated myelin damage. These findings align with previous reports indicating that PARP-1 activation exacerbates oligodendrocyte death through NAD^+^ depletion and promotion of cell death pathways, such as parthanatos [14,33]. PJ34 inhibition of PARP-1 may thereby protect against oligodendrocyte loss and, consequently, demyelination [34,35].

The therapeutic effects of PJ34 observed in this study mirror recent advances suggesting the modulation of PARP activity as a viable strategy to reduce neuroinflammation. Notably, studies in experimental autoimmune encephalomyelitis (EAE) models have shown that genetic deletion or pharmacological inhibition of PARP-1 reduces severity of symptoms, demyelination, and glial activation [36,37]. Furthermore, PARP inhibitors such as 4-ANI have shown promise in preclinical models of neurodegeneration by preserving mitochondrial function, reducing oxidative stress, and mitigating inflammatory cascades [38]. These findings support the concept that PARP-1 is a critical regulator of neuroinflammation. However, our study extends this evidence by using the LPC focal demyelination model, which allows precise spatiotemporal characterization of lesion-associated inflammation. Unlike EAE, which models systemic autoimmunity, the LPC model provides a controlled focal lesion in the brain that can be followed longitudinally. Given the non-invasive nature of PET imaging and the availability of PARP inhibitors, this work may lay the groundwork for future translational studies in MS patients.

While our study offers compelling evidence for the potential therapeutic benefit of PARP inhibitors like PJ34 in MS, it is not without limitations. The LPC model, although highly reproducible, primarily induces focal demyelination through toxin-mediated oligodendrocyte injury and does not capture the full immunological or neurodegenerative complexity of MS. This is particularly relevant given the ongoing debate as to whether MS should be regarded predominantly as an autoimmune disorder, a neurodegenerative disease, or a condition with overlapping features of both. Unfortunately, no single animal model can fully recapitulate the complexity and heterogeneity of human MS. While the LPC model is highly valuable for dissecting focal demyelination and neuroinflammation, it represents only part of the disease spectrum. Similarly, EAE and other models each emphasize different aspects of MS pathophysiology. Ultimately, the true therapeutic potential of PARP inhibitors will need to be established in clinical studies, where the complexity of immune, neurodegenerative, and reparative processes can be evaluated in an integrated manner. Diagnostic methods that can easily be translated from animals to patients, like [^11^C]PBR28 PET imaging, could be a very useful tool for this purpose. Additionally, the precise molecular mechanisms by which PARP-1 inhibition alters microglial polarization and oligodendrocyte survival remain to be elucidated. Another important question is whether PJ34 promotes remyelination, in addition to preventing demyelination. Future studies should move beyond preventive paradigms to evaluate the therapeutic potential of PJ34 when pathological processes are already underway. Initiating treatment at later time points after lesion induction would provide important insight into its curative efficacy, which is more relevant to clinical scenarios. In addition, longitudinal investigations of remyelination — including oligodendrocyte lineage dynamics and quantitative measures of myelin density and integrity — will help to determine whether PJ34 promotes repair as well as reducing inflammation. Although [^11^C]PBR28 PET already provides an indirect readout that is closely tied to mitochondrial status, it does not directly resolve how PARP inhibition affects mitochondrial metabolism. PARP-1 overactivation consumes NAD^+^ and drains cellular ATP, thereby disrupting mitochondrial energy production and shifting cells toward a state of bioenergetic failure. Given that mitochondrial dysfunction is increasingly recognized as a central driver of axonal degeneration in MS, future studies should directly examine how PARP inhibition modulates mitochondrial respiration and energy homeostasis.

In conclusion, our study demonstrates that PJ34 significantly reduced lesion-associated glial activation and attenuated demyelination in an LPC-induced rat model of focal white matter injury. Longitudinal [^11^C]PBR28 PET sensitively detected treatment-associated changes concordant with postmortem analysis, supporting its value as a noninvasive biomarker of lesion activity. Together, these findings support further investigation of PARP inhibition as a potential therapeutic strategy in demyelinating disease and highlight [^11^C]PBR28 PET as a useful translational tool for monitoring treatment response.

## CRediT authorship contribution statement

**Ge Li:** Conceptualization, Methodology, Formal analysis, Investigation, Data curation, Writing - original draft, Visualization. **Zihe Ming:** Investigation, Validation. **Chunhui Wu:** Investigation, Validation. **Inês F. Antunes:** Conceptualization, Writing - review & editing, Supervision. **Bart Cornelissen:** Conceptualization, Writing - review & editing, Supervision. **Erik F. J. de Vries:** Conceptualization, Writing - review & editing, Supervision, Project administration.

## Data availability

Data will be made available on request.

## Declaration of interests

The authors declare that they have no known competing financial interests or personal relationships that could have appeared to influence the work reported in this paper.
