# Supplementary material for: PARP inhibition attenuates neuroinflammation and demyelination in a rat model of multiple sclerosis: A longitudinal [11C]PBR28 TSPO PET imaging study
Source: Neurotherapeutics. 2026 Aug 14;23(5):e01044. doi: 10.1016/j.neurot.2026.e01044 (PMC13503139; doi:10.1016/j.neurot.2026.e01044)
Supplement: Supplementary file 1 [file mmc1.docx]

**Supplemental**

Radiochemistry

[^11^C]CH_3_I was prepared from cyclotron-produced [^11^C]CH_4_ via a gas phase reaction with iodine vapor at 700 °C. [^11^C]CH_3_I was trapped in 0.3 mL anhydrous DMSO containing 0.5 mg of DESmethyl-PBR28 and 10 mg KOH. The reaction mixture was then heated at 80°C for 4 min. After cooling down, the reaction mixture was neutralized with 0.7 mL 1M HCl and diluted with 1 ml of eluents. The resulting solution was purified by reversed phase HPLC (Waters μBondpak™ C18 125 10 μM 7.8x300mm, eluents: acetonitrile/water (43/57 *v*/v), flow: 5 mL/min). The radioactive product ([¹¹C]PBR28) with a retention time of approximately 14 min was collected in 70 ml of water. The product was transferred to an Oasis HLB (30 mg, 1cc) cartridge. The cartridge was washed twice with 8 ml of water and eluted with 0.75 ml of ethanol and 5 ml of saline. The eluate was sterilized over a 0.22 μm LG sterilization filter and collected in a sterile vial. The identity and purity of the final product were determined by analytical UPLC (UPLC BEH Phenyl 1.7µm (3x50mm), eluents: acetonitrile/water (40/60 *v*/v), flow: 0.6 mL/min). The product was isolated with an average molar activity of 129 ± 37 GBq/μmol and a radiochemical purity of 100% (n=16). For in-vitro and animal experiments, the product solution was further diluted with saline.

*Fig. S1 Iba-1 immunohistochemical staining of sections of the contralateral hemisphere of rat brain collected at 3 and 7 days post-LPC injection. Scale bars: 100 μm*

**Table. S1** [¹¹C]PBR28 PET SUV in ipsilateral LPC lesion and contralateral control regions of vehicle- and PJ34-treated rats at days 3 and 7 after LPC injection.

**Table S1A.** Individual [^11^C]PBR28 PET SUV_mean_ in the ipsilateral LPC lesion and mirrored contralateral control region.

| **Animal ID** | **Treatment** | **Day** | **Ipsilateral lesion SUV_mean_** | **Contralateral SUV_mean_** | **SUV ratio** |
| --- | --- | --- | --- | --- | --- |
| 101 | Vehicle | 3 | NA | NA | NA |
| 101 | Vehicle | 7 | 1.3084 | 0.5088 | 2.572 |
| 102 | Vehicle | 3 | 1.8459 | 0.5022 | 3.676 |
| 102 | Vehicle | 7 | 1.2660 | 0.5043 | 2.510 |
| 103 | Vehicle | 3 | 1.7294 | 0.5054 | 3.422 |
| 103 | Vehicle | 7 | 1.2242 | 0.5097 | 2.402 |
| 104 | Vehicle | 3 | 1.7516 | 0.5026 | 3.485 |
| 104 | Vehicle | 7 | 1.2374 | 0.5118 | 2.418 |
| 105 | Vehicle | 3 | NA | NA | NA |
| 105 | Vehicle | 7 | 1.2946 | 0.5071 | 2.553 |
| 106 | Vehicle | 3 | NA | NA | NA |
| 106 | Vehicle | 7 | NA | NA | NA |
| 107 | Vehicle | 3 | 1.7500 | 0.5088 | 3.439 |
| 107 | Vehicle | 7 | 1.2645 | 0.5064 | 2.497 |
| 108 | Vehicle | 3 | 1.7663 | 0.5028 | 3.513 |
| 108 | Vehicle | 7 | 1.4070 | 0.4977 | 2.827 |
| 109 | Vehicle | 3 | 1.7553 | 0.5108 | 3.436 |
| 109 | Vehicle | 7 | NA | NA | NA |
| 110 | Vehicle | 3 | 1.7273 | 0.5065 | 3.410 |
| 110 | Vehicle | 7 | NA | NA | NA |
| 111 | Vehicle | 3 | 1.7012 | 0.5011 | 3.395 |
| 111 | Vehicle | 7 | NA | NA | NA |
| 201 | PJ34 | 3 | NA | NA | NA |
| 201 | PJ34 | 7 | 1.0046 | 0.5093 | 1.973 |
| 202 | PJ34 | 3 | NA | NA | NA |
| 202 | PJ34 | 7 | 1.0007 | 0.5023 | 1.992 |
| 203 | PJ34 | 3 | 1.1991 | 0.5119 | 2.342 |
| 203 | PJ34 | 7 | 1.0013 | 0.5068 | 1.976 |
| 204 | PJ34 | 3 | 1.2493 | 0.5097 | 2.451 |
| 204 | PJ34 | 7 | 0.9987 | 0.5015 | 1.991 |
| 205 | PJ34 | 3 | NA | NA | NA |
| 205 | PJ34 | 7 | 1.0469 | 0.5065 | 2.067 |
| 206 | PJ34 | 3 | 1.1731 | 0.5014 | 2.340 |
| 206 | PJ34 | 7 | NA | NA | NA |
| 207 | PJ34 | 3 | 1.1987 | 0.5038 | 2.379 |
| 207 | PJ34 | 7 | 0.9753 | 0.5068 | 1.924 |
| 208 | PJ34 | 3 | 1.2503 | 0.5085 | 2.459 |
| 208 | PJ34 | 7 | 0.9674 | 0.5070 | 1.908 |
| 209 | PJ34 | 3 | 1.2311 | 0.5057 | 2.434 |
| 209 | PJ34 | 7 | NA | NA | NA |
| 210 | PJ34 | 3 | 1.2151 | 0.5029 | 2.416 |
| 210 | PJ34 | 7 | NA | NA | NA |
| 211 | PJ34 | 3 | 1.1743 | 0.5054 | 2.324 |
| 211 | PJ34 | 7 | NA | NA | NA |

*SUV ratio = ipsilateral lesion SUV_mean_ / contralateral SUV_mean_. NA indicates that no evaluable PET scan was available.*

**Table S1B.** [^11^C]PBR28 PET SUV_mean_ statistical results.

| **Region** | **Time point / comparison** | **Vehicle** | **PJ34** | **p-value: Vehicle vs PJ34** |
| --- | --- | --- | --- | --- |
| Ipsilateral LPC lesion | Day 3 | 1.75 ± 0.04 | 1.21 ± 0.03 | <0.001 |
| Ipsilateral LPC lesion | Day 7 | 1.28 ± 0.06 | 0.99 ± 0.03 | <0.001 |
| Ipsilateral LPC lesion | p-value: Day 3 vs Day 7 | <0.001 | <0.001 |  |
| Contralateral control | Day 3 | 0.505 ± 0.003 | 0.506 ± 0.004 | 0.530 |
| Contralateral control | Day 7 | 0.507 ± 0.005 | 0.506 ± 0.003 | 0.702 |
| Contralateral control | p-value: Day 3 vs Day 7 | 0.554 | 0.308 |  |

*Fig. S2 Representative Luxol Fast Blue (LFB)–stained coronal brain sections from rats collected at 3 and 7 days post-LPC injection. Scale bar: 500 μm. Images are representative of n = 5 per group.*

*Fig. S3 PARP1 protein expression in RAW264.7 macrophages. Western blot analysis detected full-length PARP1 at the expected molecular weight of approximately 116 kDa in three independently cultured RAW264.7 cell batches, with two replicate wells analyzed per batch. β-actin (approximately 42 kDa) served as the loading control. All six samples were analyzed on the same membrane. Lanes 1–2, biological replicate 1; lanes 3–4, biological replicate 2; and lanes 5–6, biological replicate 3.*
